# Comparison of Gene and Protein Expressions in Rats Residing in Standard Cages with Those Having Access to an Exercise Wheel

**DOI:** 10.1155/2014/950516

**Published:** 2014-02-25

**Authors:** Helaine M. Alessio, Hayden Ansinelli, Caitlyn Threadgill, Ann E. Hagerman

**Affiliations:** ^1^Department of Kinesiology and Health, Miami University, Oxford, OH 45056, USA; ^2^Department of Chemistry and Biochemistry, Miami University, Oxford, OH 45056, USA

## Abstract

Lifelong physical inactivity is associated with morbidity in adulthood, possibly influenced by changes in gene and protein expressions occurring earlier in life. mRNA (Affymetrix gene array) and proteomic (2D-DIGE MALDI-TOF/MS) analyses were determined in cardiac tissue of young (3 months) and old (16 months) Sprague-Dawley rats housed with no access to physical activity (SED) versus an exercise wheel (EX). Unfavorable phenotypes for body weight, dyslipidemia, and tumorogenesis appeared more often in adult SED versus EX. No differentially expressed genes (DEGs) occurred *between* groups at 3 or 16 months. *Within* groups, SED and EX shared 215 age-associated DEGs. In SED, ten unique DEGs occurred with age; three had cell adhesion functions (fn1, lgals3, ncam2). In EX, five unique DEGs occurred with age; two involved hypothalamic, pituitary, and gonadal hormone axis (nrob2, xpnpep2). Protein expression involved in binding, sugar metabolic processes, and vascular regulation declined with age in SED (KNT1, ALBU, GPX1, PYGB, LDHB, G3P, PYGM, PGM1, ENOB). Protein expression increased with age in EX for ATP metabolic processes (MYH6, MYH7, ATP5J, ATPA) and vascular function (KNT1, ALBU, GPX1). Differences in select gene and protein expressions within sedentary and active animals occurred with age and contributed to distinct health-related phenotypes in adulthood.

## 1. Introduction

Major advances in biomedical research and human health and disease have occurred in large part through animal experiments. The shorter life expectancy of rats (~2 years) provides an advantage when studying age-related changes that occur over the lifespan [1, 2]. The human and rat genome are very similar, both containing 25,000–30,000 protein-coding genes [3, 4], although most gene functions (~95%) are unknown [5]. Thus far, approximately 80% of rat genes have been reported to have ortholog (genes that evolved from a common ancestral gene) counterparts in humans [6] and, to date, less than 5% of genes in the rat genome have been determined to lack an analogous gene in the human genome. Additionally, almost every human gene discovered to be associated with disease has an ortholog in the rat genome. Despite the biological similarities, outcomes of animal research usually do not account for molecular or physiological alterations that may occur over the life span due to extremely low physical activity levels in laboratory animals used in research [7, 8]. Laboratory rats housed in standard 0.02 m^3^ cages move on average 160 m·day^−1^, compared with over 10,000 m·day^−1^ in animals that have access to a running wheel [9]. This very low activity coupled with ad libitum food access, likely contributed to findings from previous studies of unhealthy phenotypes including excessive weight gain, tumor development, abnormal blood lipids, hypertension, oxidative stress, and poor spatial maze performance in animals having no access to physical activity outside of a standard cage compared to those with access to an exercise wheel [9–14]. In these studies documenting phenotype differences after lifelong physical inactivity or activity, neither microarray nor proteomics was examined.

In this study, mRNA and protein expression in young and old animals confined to standard cages were compared with age-matched animals that voluntarily exercised on an exercise wheel placed inside the cages. Health-related phenotypes in these same animals were previously reported [9, 10] with most health risk traits developing so gradually, that they did not appear clinically relevant until adulthood. It is possible that ephemeral shifts in gene or protein expressions that occur throughout the life span may provide a blueprint for age-associated disease phenotypes evident in adulthood. We know of no studies that have investigated both global gene and protein expression in cardiac tissue in laboratory animals with and without access to regular physical activity. It would be useful to understand if any genes and proteins express differently in animals that are housed solely in a cage compared with animals with access to an exercise wheel, given the phenotype data in adult rats already reported.

Microarray gene analyses provided identification at the transcriptome level of genes that were over- or underexpressed comparing cardiac tissue of young and old animals with very different daily physical activity levels. Proteomic analysis provided protein abundance in cardiac tissue. Over the course of a typical lifespan, the accumulation of exercise-induced effects on genes has been shown to offset many age-related gene expression increases reported in cardiac tissue of sedentary mice [15]. Bronikowski's study reported an overall downregulation of expression for genes involved with inflammation and stress responses in a regular exercise compared with a sedentary group. It is possible that limiting regular physical activity in laboratory animals by the confines inherent in standard cages affects the expression of a number of genes and proteins in critical networks, such as inflammation and stress, which are associated with age- and sedentary-related phenotypes. In this study, gene expressions, functional networks, protein levels, and clusters were compared in 3-month- and 16-month-old male Sprague-Dawley rats that were either chronically inactive due to being housed solely in a cage or were regularly active by voluntarily running on an exercise wheel located inside the cage.

## 2. Materials and Methods

Male Sprague-Dawley rats (*N* = 24; age = 3 wk; Charles River, Wilmington, MA) were divided into two groups consisting of animals with similar mean starting weights: (1) SED (*n* = 12): resided in pairs in standard cages with no access to a running wheel throughout the study; mean starting weight = 63.6 ± 1 g, and (2) EX (*n* = 12): resided in pairs in standard cages for 24 hours and then were placed individually in a different standard sized cage equipped with a running wheel for 24 hours, with cage switching repeated every day throughout the study; mean starting weight = 59.4 ± 1 g. This schedule provided EX animals regular access to physical activity on a running wheel while controlling for the effect of paired versus individual housing. Animals were housed in standard polypropylene cages (0.454 m × 0.238 m × 0.200 m) equipped with corn cob bedding (Bed-O-Cobs), in climate-controlled rooms (24 ± 2°C) with a 12-h light/dark cycle in a normal day cycle, and free access to water and food (Purina Lab Diet 5001 Rodent Chow). The study was conducted in accordance with ethical procedures and policies and was approved by the Institutional Animal Care and Use Committee.

### 2.1. Activity and Exercise Protocols and Measurements

Handling procedures were the same for all animals and described previously [9, 10]. SED animals resided in pairs in standard cages with no items added for enrichment. EX animals resided in pairs in the same standard cages and then were placed in standard cages equipped with running wheels every other day. This provided both groups with socialization as well as regular access to a running wheel for the EX group. Animal movement in the cages was monitored by a 24-hour surveillance VDI-2000BI B/W CCD IR camera, using infrared light. Two animals from each group were randomly selected each month and then marked with a permanent marking pen for tracking purposes. Recorded videos were evaluated by two individuals trained to trace movement inside the cage following the marked animals. Daily distance in SED was determined by averaging both total distance measures covered in 24 hours and recorded in meters. Daily distance in EX was determined by wheel revolutions converted to meters·day^−1^ in each EX animal.

SED and EX rats were sacrificed at 3 mo and 16 mo in a rested condition by decapitation and exsanguination. Serum was collected and separated following centrifugation (2000 ×g for 15 min). Organs and tissues were harvested immediately and were deep-frozen at −80°C for RNA and protein isolation described below.

### 2.2. Gene Expression Analyses

Global gene expression was measured using RNA from the frozen heart tissue. Briefly, RNA was extracted from 20–30 mg samples cut from the left ventricles using Qiagen RNeasy (Valencia, CA) RNA extraction kits. The absorbance ratios (A260/A280) were determined using a ND-1000 UV/VIS spectrophotometer (Nanodrop Technologies, Inc., Montchanin, DE) and were in a range from 2.04 to 2.17. The integrity of each sample was further assessed with an Agilent BioAnalyzer system by the Biomedical Genomics Core at the Research Institute at Nationwide Children's Hospital (Columbus, OH) and determined to be of high quality before processing. All samples passed quality control and were pooled within each treatment group to yield 4 gene chips per treatment group, with RNA from 3 animals on each chip, to ensure correct quantity of total RNA. The pooling of RNA samples allowed for representation in a cost-effective analysis. Samples were labeled with the Affymetrix Whole Transcript Labeling system and then hybridized to the Affymetrix GeneChip Rat Gene 1.0 ST Array (Santa Clara, CA). The design of the Rat Gene 1.0 ST Array is based primarily on a subset of GeneChip Rat Exon 1.0 ST Array probes that map to well-supported exons of known genes. The array comprised more than 700,000 unique 25-mer oligonucleotide features constituting more than 27,000 gene-level probe sets. Data was preprocessed using the RMA approach for background correction, normalization, and probe set summarization using the Bioconductor affy package in R.

Significance Analysis of Microarrays (SAM) used the Bioconductor Siggenes package to identify differentially expressed genes (DEGs) between 3 mo and 16 mo old animals for each treatment group. A two-class unpaired analysis with a false discovery rate (FDR) of 10% was used to maximize sensitivity and minimize the effect on accuracy. Comparisons were made between 3 mo EX versus 3 mo SED and between 16 mo EX versus 16 mo SED. Microarray results are available in the Gene Expression Omnibus (GEO) database (http://www.ncbi.nlm.nih.gov/geo/) under the GEO accession no. GPL6247. Samples can be located with GEO accession nos. GSM1244311, GSM1244312, GSM1244313, GSM1244314, GSM1244319, GSM1244320, GSM1244321, GSM1244322, GSM1244335, GSM1244336, GSM1244337, GSM1244338, GSM1244343, GSM1244344, GSM1244345, and GSM1244346.

2-dimensional difference gel electrophoresis (2D-DIGE), matrix-assisted laser desorption/ionization time-of-flight mass spectrometry (MALDI-TOF MS) and protein cluster analyses identification were performed by Applied Biomics, Inc. (Hayward, CA). A small portion (10 mg) of cardiac tissue from the left ventricle of each animal was washed with 10 mM Tris-HCl, 5 mM magnesium acetate, pH 8.0 three times to remove any contaminating blood. Then 200 *μ*L of 2D cell lysis buffer (30 mM Tris-HCl, pH 8.8, containing 7 M urea, 2 M thiourea, and 4% CHAPS) was added to the tissue. The mixture was sonicated at 4°C followed by shaking for 30 min at room temperature and centrifugation for 30 min at 21,000 g. Protein concentration in the supernatants was measured using Bio-Rad protein assay method.

For each cardiac sample, 30 *μ*g of protein was mixed with 1.0 *μ*L of diluted CyDye and kept in the dark on ice for 30 min. A protocol described by Berkelman and Stenstedt [16] was followed. Samples from each pair were labeled with Cy3 and Cy5 dyes, respectively. An internal standard was made up of equal amounts of protein from each sample and labeled with Cy2 and run on every gel. So, in each gel, there were 3 samples that were individually labeled: an internal standard labeled with Cy2, the first sample labeled with Cy3, and the second sample labeled with Cy5. The labeling reaction was stopped by adding 1.0 *μ*L of 10 mM lysine to each sample and incubating in dark on ice for additional 15 min. The labeled samples were then mixed together. The 2X 2D sample buffer (8 M urea, 4% CHAPS, 20 mg/mL dithiothreitol (DTT), 2% pharmalytes, and trace amount of bromophenol blue), 100 *μ*L destreak solution, and rehydration buffer (7 M urea, 2 M thiourea, 4% CHAPS, 20 mg/mL DTT, 1% pharmalytes, and trace amount of bromophenol blue) were added to the labeling mix to bring the total volume to 250 *μ*L before loading the labeled samples into the strip holder.

After loading the labeled samples, isoelectric focusing (IEF) was run following the protocol provided by Amersham BioSciences. Upon finishing the IEF, the immobilized pH gradient (IPG) strips were incubated in the freshly made equilibration buffer-1 (50 mM tris-HCl, pH 8.8, containing 6 M urea, 30% glycerol, 2% sodium dodecyl sulfate (SDS), trace amount of bromophenol blue, and 10 mg/mL DTT) for 15 minutes with gentle shaking. Then the strips were rinsed in the freshly made equilibration buffer-2 (50 mM Tris-HCl, pH 8.8, containing 6 M urea, 30% glycerol, 2% SDS, trace amount of bromophenol blue, and 45 mg/mL DTT) for 10 min with gentle shaking. Next the IPG strips were rinsed in the SDS-gel running buffer before transferring into 12% SDS-gels. The SDS-gels were run at 15°C until the dye front reached the end of the gels.

Gel images were scanned immediately following the SDS polyacrylamide gel electrophoresis (SDS-PAGE) using Typhoon TRIO (GE Healthcare). Scanned images were then analyzed by Image Quant software (version 6.0, GE Healthcare), followed by cross-gel analysis using DeCyder software (version 6.5, GE Healthcare). Fold changes of the protein expression levels were obtained from in-gel DeCyder analysis. Specific proteins were identified by matrix assisted laser desorption/ionization time of flight mass spectrometry (MALDI-TOF/MS) and meeting criteria of 1.3-fold and *P* < 0.05 comparing old and young in SED and EX groups and correlated with protein spot numbers with an altered density. A follow-up completed the cluster pathway analysis, using a public bioinformatics tool available from the National Institutes of Health (NIH) called The Database for Annotation, Visualization and Integrated Discovery (DAVID).

DAVID, a public bioinformatics tool available from the NIH, was used to explore functionality and meaning of the list of proteins entered into the analyses [17]. All proteins entered into the analysis were first annotated into >40 annotation categories or clusters, including GO terms, protein-protein interactions, protein functional domains, disease associations, biopathways, sequence general features, homologies, gene functional summaries, and gene tissue expressions. DAVID software uses a novel algorithm to measure the relationships among the annotation terms based on the degrees of their coassociation genes to group the similar, redundant, and heterogeneous annotation contents from the same or different resources into annotation groups.

## 3. Results

### 3.1. Daily Physical Activity of SED and EX Groups

Distance covered in 24 hours for both SED and EX animals peaked at 2 mo of age and then, as demonstrated previously [9, 10], declined with age. However, the difference in daily physical activity between the two groups was stark. Mean daily distance of 161 ± 26 m·day^−1^ in the SED group was consistent over the life span. In contrast, animals in the EX group had a mean peak distance of 5548 ± 273 m·day^−1^ at 2 mo old on the running wheel. When housed in the standard cage with no exercise wheel every other day EX animals covered only 111 ± 10 m·day^−1^. When in the cage equipped with a running wheel, daily activity other than wheel running was negligible and averaged 45 m·day^−1^.

**Figure 1:**
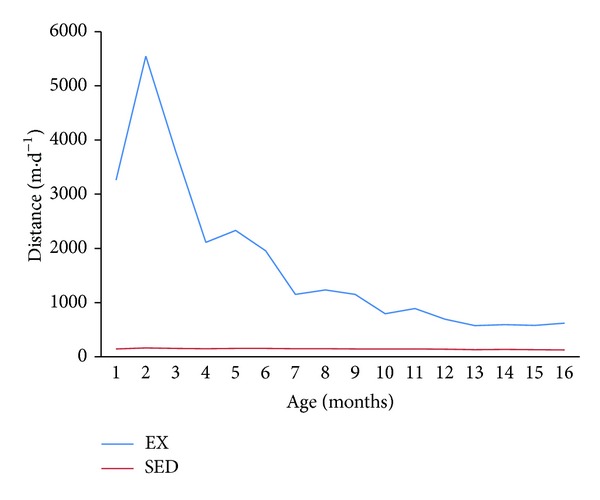
Mean daily distance covered (m·d^−1^) in rats over time that were housed in standard cages (SED) and rats when housed individually in standard cages equipped with running wheels (EX).

With increasing age, animals in the EX group became less active when housed in the cage with the running wheel, as shown by mean distances that decreased after 2 months with a peak of 5,548 m·day^−1^ to approximately 2000 m·day^−1^ at 6 mo and 500–1000 m·day^−1^ between 6 and 16 mo (Figure 1). Exercise intensity was not measured in this study; however, previous reports indicated that regular wheel running reduced body mass, visceral fat, and positively impacted lipid and amino acid metabolism [18–20], while not affecting oxidative capacity in rat muscle. We previously reported heart : body weight ratios as 0.370 ± 0.007 in EX and 0.340 ± .0007 in SED, which were not found to reach statistical significance. The main indicators that regular physical activity of the EX group resulted in physiological differences compared with the SED group have been previously reported [9, 10], and included EX having a significantly lower body weight throughout the life span with final mean body weights of 683 ± 14 versus 737 ± 18 g, lower prolactin levels (4.2 ± 0.6 versus 9.9 ± 2.4 ng·mL^−1^), a more favorable oxidative stress balance represented by GSH : GSSG (189 versus 117), and fewer tumors (54% fewer) which were all benign cysts in EX compared with more tumors, including thyroid carcinoma and malignancy in SED.

### 3.2. Microarray Gene Expression

Microarray results comparing SED toEX at 16 mo and 3 mo showed no effect of exercise treatment, controlling for age, with no differentially expressed genes (DEGs). This surprising finding indicated that gene expression for animals that exercised on a running wheel was indistinguishable from gene expression for their sedentary counterparts of the same age. Phenotype changes that were obvious at 16 months in the absence of gene expression differences *between* groups may be the result of a relatively small number of modified gene expressions that occurred briefly, earlier in life. These gene expressions may have initiated the translation of key health-related proteins and then returned to a baseline transcription level. For animals in either group, there were unique changes in gene expression with age, supporting the hypothesis that exercise exerts an effect on gene expression early in life, even though physiological consequences may not be apparent until late in life. There was a significant age effect *within* SED and EX groups (Table 1) and a comparison of gene expressions in 16 mo and 3 mo old rats within SED and EX resulted in 228 DEGs in SED and 230 DEGs in EX, with 215 of those gene expressions shared by both groups. Genes with the largest disparate expressions between young and old in SED and EX were involved in vascular function, homeostasis, oxidative stress, and cholesterol (e.g., Col3a1, Emb, Atp2b2, Fmo1, Cyp2e1, Nox4, InhA, Chrna1, and Cyp1a1).

Table 2 displays the names of common genes expressed at least 2-fold differently between 16 mo and 3 mo old animals in SED or EX groups. The four largest DEGs occurred for the following genes: Atp2b2 (functions in intracellular calcium homeostasis), Cyp1a1 (roles in drug metabolism, synthesis of cholesterol, steroids, lipids, and NADPH-dependent electron transport pathway), Chrna1 (functions in acetylcholine binding/channel gating), and Col3a1 (roles in connective tissue and vascular function). None of these genes have previously been associated with physical activity, although Col3a1 was suspected to be associated with muscle cramping [21].  Table 3 displays fifteen unique DEGs with fold changes of at least 1.5-fold between 16 mo and 3 mo old animals in both groups. Ten unique DEGs were identified for SED, with three having cell adhesion functions (e.g. Fn1, Lgals3, and Ncam1). Only five unique genes were associated with EX, with two involved in the hypothalamic, pituitary, and gonadal hormone axis and obesity (e.g., Nrob2 and Xpnpep2). Candidate genes that were previously associated with voluntary exercise, including glucose transporter 4 (Glut4) [22], nescient helix loop helix 2 (Nhlh2) [23], and dopamine receptor 1 (Drd1) [24], were not identified as DEGs in the current study.

In a review of gene candidates that regulate physical activity, Lightfoot [25] describes a strong case for two candidate genes, Drd1, which regulates dopamine levels, and Nhlh2, which affects B-endorphin levels. Both gene candidates meet four standards of evidence from research including (1) having functional relevance to a known trait, (2) localizing within an identified quantitative trait loci, (3) having a possible genomic structural variation in the gene that may give rise to a functional difference in a protein, and (4) demonstrating a difference in gene expression as well as a difference in trait. Lightfoot states that other genes are likely to emerge as strong candidates, recognizing that epigenetic forces, including acetylation and methylation, may also be strong regulators of traits associated with physical activity. Furthermore, combinations of genes that are up- and downregulated can communicate in networks that ultimately translate for proteins that regulate cell function. In the current study, the largest DEGs expressed with age in both SED and EX were Atp2b2 (SED: 1.7 versus EX: −8.9) and Col3a1 (SED = −1.5 versus EX: +3.6). Other DEGs, whether shared or unique in SED and EX, were approximately 2-fold different or less. These data suggest resilience in gene expression over time, with only a small number of genes modified as a result of either chronic inactivity or activity.

### 3.3. 2D-DIGE, SDS PAGE, and MALDI-TOF-MS

**Figure 2:**
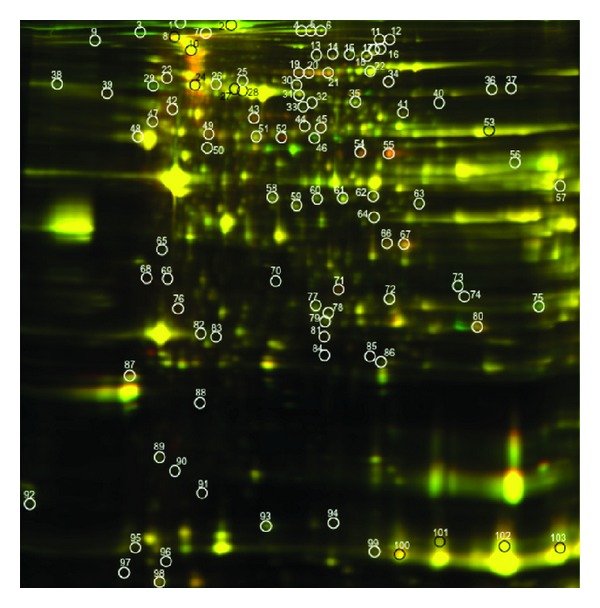
2-dimensional gel electrophoresis image of overlay comparing 3-month-old SED and 3-month-old EX animals. Proteins (*n* = 103) showing different expressions (>1.5, *P* < 0.05) are circled.

**Figure 3:**
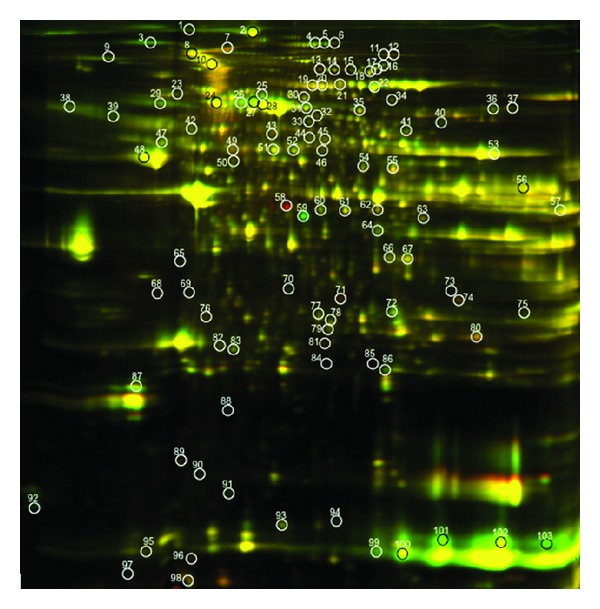
2-dimensional gel electrophoresis image of overlay comparing 16 mo old SED and 16 mo old EX animals. Proteins (*n* = 103) showing different expressions (>1.5, *P* < 0.05) are circled.

**Figure 4:**
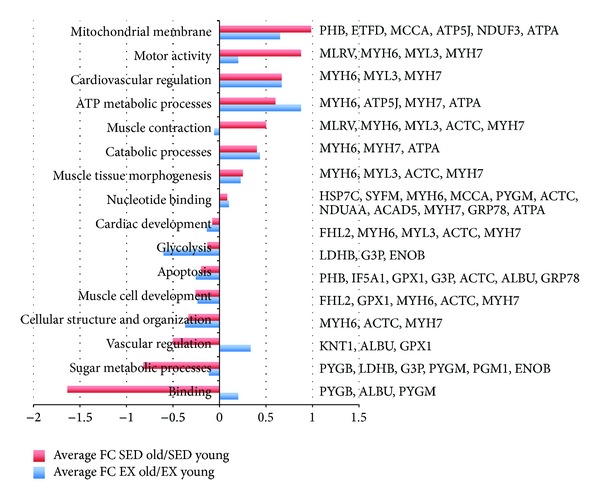
Functional category, protein names, and fold changes in SED and EX, old and young, FC = fold change.

2D-DIGE and SDS PAGE distinguished 103 proteins that met statistical significance when comparing old and young animals in SED and EX groups. Of the 103 spots, 58 of these proteins were identified by MALDI-TOF-MS (Figures 2 and 3). Significant spots on the gel were identified independently in each comparison, and the number of significant spots varies by comparisons. Some spots could be significant in multiple comparisons. Supplementary Table  1 (see Supplementary Material available online at http://dx.doi.org/10.1155/2014/950516) lists the names of the DIGE-identified proteins in cardiac tissue that changed in abundance, identified by MALDI-TOF/MS and meeting criteria of 1.3-fold and *P* < 0.05, comparing old and young in SED and EX groups, and correlated with protein spot numbers with altered densities in Figures 2 and 3, respectively. Relevant information about the match quality, molecular weight, isoelectric point, protein scores, and confidence levels is provided in Supplementary Table  1. Supplementary Table  2 lists protein spot numbers that correspond to the names in Supplementary Table  1. Also listed are the comparative ratios in SED old versus young and EX old versus young, along with their *P* values. Fold changes in specific proteins, as well as functional categories in old and young animals in both SED and EX are shown in Figure 4.

Results of the DAVID bioinformatics cluster analyses showed that, compared with EX, SED had higher proteins levels in old versus young that were associated with mitochondrial membrane, motor activity, and muscle contraction categories and lower protein levels for binding, sugar metabolic processes, and vascular regulation (e.g., KNT1, ALBU, GPX1, PYGB, LDHB, G3P, PYGM, PGM1, and ENOB). Compared with SED, the EX group had higher protein levels in only two functional categories: ATP metabolic processes (e.g., MYH6, MYH7, ATP5J, and ATPA) and vascular function (e.g., KNT1, ALBU, and GPX1).

## 4. Discussion

Global gene expression and 2D-DIGE were combined in this study to compare gene and protein expressions in physically inactive animals that resided solely in a standard cage with physically active animals having regular access to an exercise wheel. Exercise training-induced changes in contractile, mitochondrial, and transporter protein expression in rat skeletal muscle have been previously described [26, 27]. Yamaguchi et al. [28] identified 13 protein expression differences between high-intensity exercise trained and sedentary animals, several of which had previously unknown functions related to regular exercise. Several studies have reported that 65–73% of the variance in protein abundance may be explained by mRNA concentration [29, 30]; however, due to miRNA interactions, posttranslational modifications protein degradation, and sometimes unpredictable protein response to stimuli, mRNA alone is not always a good predictor of protein abundance [29, 30].

Although no DEGs were observed when comparing same-aged animals in different activity treatment groups, 2D-DIGE and SAGE analyses revealed 103 proteins at different measurable levels in 16 mo old SED versus 16 mo old EX rats and 3 mo SED versus 3 mo old SED rats. Comparing EX and SED, similar age-associated protein abundances were found for ETFD, HBB1, APOA1, MLRV, SPA3K, and FHL2 in both groups, indicating a more powerful age rather than physical activity influence on these protein expressions. Proteins that were expressed differently in old versus young SED compared with old versus young EX, indicating a more powerful influence of physical activity on protein expression, included NDUF3, ATP5J, G3P, GPX1, HSP7C, MCCA, PYGB, IDH3B, PYGM, ALDH2, ACADS, LDHB, MYL3, ALBU, and TTHY. These proteins represent a small fraction of the total number of the tens of thousands of proteins estimated in the entire organism. In our investigation, health-related differences between exercised and sedentary animals were not clinically obvious until the rats were 12 months old. After this age, animals housed in standard cages without access to a running wheel, exhibited undesirable phenotypes such as high blood cholesterol, high body weight, hypertension, greater number of tumors, and compromised spatial maze performance, compared with their more active counterparts [9, 10, 14]. The top proteins that were differentially expressed when comparing SED (old versus young) to EX (old versus young) groups (e.g., NDUF3, ATP5J, G3P, GPX1, and HSP7C) encompassed a wide variety of regulatory functions, including metabolism, oxidative stress, glycosaminoglycan binding, response to vitamin A, cell morphogenesis, activation of protein kinase activity, fatty acid biosynthesis, and contractile activity. Other proteins with large differences in expression between SED and EX had unknown functions related to exercise (e.g., four and a half LIM domains protein 2, ES1 protein homolog).

A surprisingly small number of genes and proteins have been found to be involved in cell regulation, with single proteins having multiple functions [5]. In previous studies only ~1.5% of genes from cardiac tissue in rats were differentially expressed when comparing animals at rest with those following an acute (one-time) bout of exercise [12, 27]. One study reported that sedentary female rat muscle exhibited 52 significant changes in gene transcription after one hour of vigorous exercise compared with skeletal muscle collected from resting animals [31]; however, any effect of these gene expressions on protein abundance was not investigated. In the present study, cardiac gene expressions were measured using tissue from animals sacrificed in a rested state, and so any change in expression, up or down, which may have occurred due to acute physical activity, were likely to have returned to baseline upon recovery. The small number of DEGs and proteins comparing old to young animals with different lifelong activity levels contrasted with large differences in phenotypes described previously in these animals (e.g., higher body weight, blood pressure, and tumor formation in less active) that did not emerge until early adulthood (12 months old) [9, 10].

Gene and protein expressions do not always clearly link to specific health- or disease-associated phenotypes. Yet the tissue-specific protein complement of the genome usually governs the function of the cell and in so doing regulates the phenotype. The emerging field of proteomics identifies and quantifies proteins that are translated, whether or not they can be predicted from DNA or mRNA analyses [28–30, 32]. The value of proteomic data ultimately depends upon its predictive ability of certain phenotypes. It is unclear if health-related phenotypes such as blood pressure, body weight, tumors, and oxidative stress can be traced to changes in a small number of genes or gene clusters, or entire networks that communicate with a small number or cluster of specific proteins.

## 5. Conclusion

The impact of environment, specifically, chronic inactivity imposed by space limitations of standard cages on gene expression and protein levels, is becoming recognized as an important consideration in animal research. Providing an exercise wheel is a simple, yet effective way to influence gene and protein expressions likely to influence health and disease traits in adulthood. Nevertheless, most animal studies neglect including information about physical activity and typically confine animals to standard cages, forcing them to be sedentary. Results from this study suggest that ephemeral changes in a small number of DEGs occur early in life and contribute to different protein abundances associated with healthy and disease traits that become evident in adulthood, differing substantively between physically inactive and inactive animals. It is currently unclear whether the predominant influence on protein synthesis stems from large changes in a small number of genes, or small changes in a large number of genes located in specific gene networks. Specific proteins expressed in lower and higher levels as a result of physical inactivity or activity are likely to affect health status, especially as one ages. Mechanisms by which lifetime physical inactivity or regular activity acts to affect the expression of specific genes and proteins remain a logical direction for future research, and the importance of housing and access to physical activity deserves further attention in future animal studies.

## Supplementary Material

Table 1 presents the analyses report for the protein-peptide by spot, location of samples on the MALDI plate, and match quality. Names of proteins are presented along with accession number, molecular weight, isoelectric point, number of matching peptides, protein score, confidence of protein identification calculated from the mass spectrophotometer (MS) data, total ion score, and total ion confidence calculated from the MS/MS data.
Table 2 presents results of fold change analyses and p-values for proteins analyzed based on assigned wells, original protein identification spots, Appearance or the number of gels out of 72 that the spots can be detected, p-values and average ratios for age-related differences within the Sedentary group and age-related differences with the Exercise group.

## Figures and Tables

**Table 1:** Number of differentially expressed genes (DEGs) comparing 16 mo and 3 mo animals residing solely in a cage (SED) or with access to an exercise wheel (EX), measured by gene array analysis on mRNA isolated from heart tissue.

Comparison group	Reference group	Total number of DEGs	Number of upregulated genes	Number of downregulated genes	Number of DEGs with >2-fold increase	>Number of DEGs with >2-fold decrease
SED 16 mo	SED 3 mo	228	123	105	23	15
EX 16 mo	EX 3 mo	230	133	97	19	20

**Table 2:** Common differentially expressed genes in SED and EX groups comparing 16 mo and 3 mo old animals.

Gene Symbol	Gene Name	Gene Function	Fold Change SED	Fold Change EX
*col3a1 *	Collagen type III alpha 1	Connective tissue and vascular function	−1.5*	3.6^#^

*emb *	Embigin	Cell growth and homeostasis	−1.7*	2.0^#^

*atp2b2 *	ATPase Calcium transporting plasma membrane 2	Intracellular calcium homeostasis	1.7^#^	−8.9*

*fmo1 *	Flavin containing monoxidase 1	Oxidative metabolism	1.8^#^	−2.2*

*cyp2e1 *	Cytochrome P450 family, 2, subfamily E polypeptide	Metabolism, cholesterol, and other lipid regulation	2.0^#^	−1.6*

*npr3 *	Natriuretic peptide receptor C/guanylate cyclase C	Regulates blood volume and pressure cardiac function, metabolism	2.0^#^	2.3^#^

*nax4 *	NADPH oxidase 4	Generates superoxide, functions as an oxygen sensor, apoptosis	−2.1*	1.5^#^

*nadph oxidase 4 *	NADPH oxidase 4	Generates superoxide, functions as an oxygen sensor, apoptosis	−2.1*	−1.7*

*inha *	Inhibin alpha	Hypothalamic, pituitary, gonadal hormone secretion, germ cell development and maturation, erythoid differentiation, insulin secretion, nerve cell survival, embryonic axial development or bone growth	−2.6*	1.7^#^

*chrna1 *	Cholinergic receptor, nicotinic, alpha 1	Plays a role in acetylcholine binding/channel gating	4.0^#^	2.3^#^

*cyp1a1 *	Cytochrome P450, family 1, subfamily A polypeptide 1	Drug metabolism and synthesis of cholesterol, steroids and other lipids, involved in an NADPH-dependent electron transport pathway	−4.1*	−1.6*

^#^: Up-regulation; *: down-regulation.

**Table 3:** Genes that exhibit unique fold changes in animals in SED and EX groups, for SED and EX, 16 mo versus 3 mo.

Gene symbol	Gene name	Gene function	Fold change SED	Fold change EX
*myoc *	Myocilin, trabecular meshwork inducible glucocorticoid response	Cytoskeletal function and is expressed in many occular tissues	1.7^#^	

*fn1 *	Fibronectin 1	Involved in cell adhesion, cell motility, opsonization, wound healing, and maintenance of cell shape	−1.5*	

*s100a10 *	S100 calcium binding protein A10	Regulator of protein phosphorylation, involved in the regulation of a number of cellular processes such as cell cycle progression and differentiation	−1.5*	

*lgals3 *	Lectin, galactoside-binding, soluble, 3	Involved in acute inflammatory responses including neutrophil activation and adhesion, chemoattraction of monocytes macrophages, opsonization of apoptotic neutrophils, and activation of mast cells	−1.6*	

*il33 *	Interleukin 33	Induces T-helper type 2-associated cytokines	1.6^#^	

*vcan *	Versican	May play a role in intercellular signaling and in connecting cells with the extracellular matrix. May take part in the regulation of cell motility, growth, and differentiation. Binds hyaluronic acid.	−1.6*	

*emp1 *	Epithelial membrane protein 1		−1.5*	

*nr1d1 *	Nuclear receptor subfamily 1, group D, member 1		−1.5*	

*ncam1 *	Neural cellular adhesion molecule	Cell adhesion molecule involved in neuron-neuron adhesion, neurite fasciculation, and outgrowth of neurites	1.6^#^	

*fhl3 *	Four and a half LIM domains 3	May be involved in tumor suppression, repression of MyoD expression, and repression of IgE receptor expression	−1.6*	

*nr0b2 *	nuclear receptor subfamily 0, group B, member 1	Component of a cascade required for the development of the hypothalamic-pituitary-adrenal-gonadal axis		−1.5*

*ptgfr *	Prostaglandin F receptor	Receptor for prostaglandin F2-alpha (PGF2alpha). The activity of this receptor is mediated by G proteins which activate a phosphatidylinositol-calcium second messenger system. Initiates luteolysis in the corpus luteum		1.8^#^

*xpnpep2 *	Inhibin alpha	Hypothalamic, pituitary, gonadal hormone secretion, germ cell development and maturation, erythroid differentiation, insulin secretion, nerve cell survival, embryonic axial development, or bone growth		1.5^#^

*abca1 *	Cholinergic receptor, nicotinic, alpha 1	Plays a role in acetylcholine binding/channel gating		1.5^#^

*pla2g2a *	Cytochrome P450, family 1, subfamily A polypeptide 1	Drug metabolism and synthesis of cholesterol, steroids, and other lipids, involved in an NADPH-dependent electron transport pathway		−1.7*

^#^: upregulation; *: downregulation.
