# Supplementary material for: Comparison of Gene and Protein Expressions in Rats Residing in Standard Cages with Those Having Access to an Exercise Wheel
Source: Biomed Res Int. 2014 Feb 25;2014:950516. doi: 10.1155/2014/950516 (PMC3955688; doi:10.1155/2014/950516)
Supplement: Supplementary file 1 — Table 1 presents the analyses report for the protein-peptide by spot, location of samples on the MALDI plate, and match quality. Names of proteins are presented along with accession number, molecular weight, isoelectric point, number of matching peptides, protein score, confidence of protein identification calculated from the mass spectrophotometer (MS) data, total ion score, and total ion confidence calculated from the MS/MS data. Table 2 presents results of fold change analyses and p-values for proteins analyzed based on assigned wells, original protein identification spots, Appearance or the number of gels out of 72 that the spots can be detected, p-values and average ratios for age-related differences within the Sedentary group and age-related differences with the Exercise group. [file 950516.f1.pdf]

Supplementary Table 1. Protein – Peptide summary identification by spot number.

Spot number	MALDI well number	Match Quality	Top Ranked Protein Name (Species)	Accession No.	Protein MW (Dalton)	Protein PI	Pep.Count	Protein Score	Protein Score C. I. %	Total Ion Score	Total Ion C. I. %
1	C1		Myosin-7 OS=Rattus norvegicus GN=Myh7 PE=2 SV=2	MYH7_RAT	222,945	5.6	31	158	100	75	100
2	C2		myomesin 2 [Rattus norvegicus]	gi|281306803	164,609	5.6	44	851	100	577	100
3	C3		Alpha-1-inhibitor 3 OS=Rattus norvegicus GN=A1i3 PE=1 SV=1	A1I3_RAT	163,670	5.7	23	282	100	201	100
5	C4		Myosin-binding protein C, cardiac-type OS=Rattus norvegicus GN=Mybpc3 PE=2 SV=2	MYPC_RAT	140,674	6.1	42	880	100	583	100
6	C5		Myosin-binding protein C, cardiac-type OS=Rattus norvegicus GN=Mybpc3 PE=2 SV=2	MYPC_RAT	140,674	6.1	22	194	100	116	100
7	C6		Myosin-6 OS=Rattus norvegicus GN=Myh6 PE=2 SV=2	MYH6_RAT	223,370	5.6	42	673	100	487	100
9	C7		Murinoglobulin-1 OS=Rattus norvegicus GN=Mug1 PE=2 SV=1	MUG1_RAT	165,221	5.7	24	543	100	450	100
13	C8		Glycogen phosphorylase, brain form (Fragment) OS=Rattus norvegicus GN=Pygb PE=1 SV=3	PYGB_RAT	96,113	6.2	31	581	100	380	100
15	C9		Glycogen phosphorylase, muscle form OS=Rattus norvegicus GN=Pygm PE=2 SV=5	PYGM_RAT	97,212	6.9	29	497	100	325	100
19	C10		Neurexophilin-4 OS=Rattus norvegicus GN=Nxph4 PE=2 SV=1	NXPH4_RAT	32,924	9.9	3	36	0	28	92
23	C11		78 kDa glucose-regulated protein OS=Rattus norvegicus GN=Hspa5 PE=1 SV=1	GRP78_RAT	72,302	5.1	8	103	100	82	100
26	C12		Heat shock cognate 71 kDa protein OS=Rattus norvegicus GN=Hspa8 PE=1 SV=1	HSP7C_RAT	70,827	5.4	19	399	100	295	100
27	C13		Serum albumin OS=Rattus norvegicus GN=Alb PE=1 SV=2	ALBU_RAT	68,686	6.1	27	692	100	530	100
28	C14		Serum albumin OS=Rattus norvegicus GN=Alb PE=1 SV=2	ALBU_RAT	68,686	6.1	26	770	100	616	100
29	C15		T-kininogen 1 OS=Rattus norvegicus GN=Map1 PE=1 SV=2	KNT1_RAT	47,745	6.1	9	394	100	354	100
33	C16		Phosphoglucomutase-1 OS=Rattus norvegicus GN=Pgm1 PE=1 SV=2	PGM1_RAT	61,365	6.3	20	247	100	120	100
34	C17		Methylcrotonoyl-CoA carboxylase subunit alpha, mitochondrial OS=Rattus norvegicus GN=Mccc1 PE=1 SV=	MCCA_RAT	79,279	6.7	12	80	100	41	100
39	C18		Serine protease inhibitor A3K OS=Rattus norvegicus GN=Serpina3k PE=1 SV=3	SPA3K_RAT	46,532	5.3	13	430	100	357	100
40	C19		Electron transfer flavoprotein-ubiquinone oxidoreductase, mitochondrial OS=Rattus norvegicus GN=Etf	ETFD_RAT	68,155	7.3	12	322	100	276	100
43	C20		Tripartite motif-containing protein 72 OS=Rattus norvegicus GN=Trim72 PE=2 SV=1	TRI72_RAT	52,798	5.9	24	821	100	649	100
50	C21		Fibrinogen gamma chain OS=Rattus norvegicus GN=Fgg PE=1 SV=3	FIBG_RAT	50,601	5.6	11	296	100	243	100
52	C22		Aldehyde dehydrogenase, mitochondrial OS=Rattus norvegicus GN=Aldh2 PE=1 SV=1	ALDH2_RAT	56,453	6.6	23	707	100	533	100
54	C23		Beta-enolase OS=Rattus norvegicus GN=Eno3 PE=1 SV=3	ENOB_RAT	46,984	7.1	19	676	100	533	100
55	C24		Beta-enolase OS=Rattus norvegicus GN=Eno3 PE=1 SV=3	ENOB_RAT	46,984	7.1	20	830	100	674	100
58	D1		NADH dehydrogenase [ubiquinone] 1 alpha subcomplex subunit 10, mitochondrial OS=Rattus norvegicus G	NDUAA_RAT	40,468	7.6	22	1070	100	876	100
59	D2		NADH dehydrogenase [ubiquinone] 1 alpha subcomplex subunit 10, mitochondrial OS=Rattus norvegicus G	NDUAA_RAT	40,468	7.6	17	1060	100	928	100
60	D3		Short-chain specific acyl-CoA dehydrogenase, mitochondrial OS=Rattus norvegicus GN=Acads PE=1 SV=2	ACADS_RAT	44,737	8.5	15	566	100	467	100
61	D4		Short-chain specific acyl-CoA dehydrogenase, mitochondrial OS=Rattus norvegicus GN=Acads PE=1 SV=2	ACADS_RAT	44,737	8.5	18	1010	100	866	100
62	D5		Isocitrate dehydrogenase [NAD] subunit beta, mitochondrial OS=Rattus norvegicus GN=Idh3B PE=1 SV=1	IDH3B_RAT	42,327	8.9	14	488	100	406	100
66	D6		Four and a half LIM domains protein 2 OS=Rattus norvegicus GN=Fhl2 PE=1 SV=1	FHL2_RAT	32,065	7.3	20	390	100	271	100
67	D7		Four and a half LIM domains protein 2 OS=Rattus norvegicus GN=Fhl2 PE=1 SV=1	FHL2_RAT	32,065	7.3	23	526	100	370	100
68	D8		Actin, alpha cardiac muscle 1 OS=Rattus norvegicus GN=Actc1 PE=1 SV=1	ACTC_RAT	41,992	5.2	13	538	100	456	100
69	D9		Prohibitin OS=Rattus norvegicus GN=Phb PE=1 SV=1	PHB_RAT	29,802	5.6	10	665	100	602	100
71	D10		Phenylalanine--tRNA ligase, mitochondrial OS=Rattus norvegicus GN=Fars2 PE=2 SV=1	SYFM_RAT	55,130	7.8	4	39	9	31	98
72	D11		Carbonic anhydrase 2 OS=Rattus norvegicus GN=Ca2 PE=1 SV=2	CAH2_RAT	29,096	6.9	13	602	100	498	100
73	D12		Glyceraldehyde-3-phosphate dehydrogenase OS=Rattus norvegicus GN=Gapdh PE=1 SV=3	G3P_RAT	35,805	8.1	11	515	100	445	100
75	D13		Electron transfer flavoprotein subunit beta OS=Rattus norvegicus GN=Etfb PE=2 SV=3	ETFB_RAT	27,670	7.6	6	453	100	426	100
76	D14		L-lactate dehydrogenase B chain OS=Rattus norvegicus GN=Ldhb PE=1 SV=2	LDHB_RAT	36,589	5.7	10	54	97		
78	D15		Myosin-6 OS=Rattus norvegicus GN=Myh6 PE=2 SV=2	MYH6_RAT	223,370	5.6	24	365	100	302	100
80	D16		ES1 protein homolog, mitochondrial OS=Rattus norvegicus PE=1 SV=2	ES1_RAT	28,155	9.1	11	763	100	681	100
81	D17		Maleylacetoacetate isomerase OS=Rattus norvegicus GN=Gstz1 PE=1 SV=2	MAAI_RAT	23,946	7.6	10	233	100	161	100
83	D18		Apolipoprotein A-I OS=Rattus norvegicus GN=Apoa1 PE=1 SV=2	APOA1_RAT	30,043	5.5	19	468	100	307	100
84	D19		ATP synthase subunit alpha, mitochondrial OS=Rattus norvegicus GN=Atp5a1 PE=1 SV=2	ATPA_RAT	59,717	9.2	10	517	100	479	100
85	D20		Glutathione peroxidase 1 OS=Rattus norvegicus GN=Gpx1 PE=1 SV=4	GPX1_RAT	22,292	7.7	7	257	100	214	100
87	D21		Myosin regulatory light chain 2, ventricular/cardiac muscle isoform OS=Rattus norvegicus GN=Myl2 PE	MLRV_RAT	18,868	4.9	13	500	100	377	100
88	D22		Myosin light chain 3 OS=Rattus norvegicus GN=Myl3 PE=1 SV=2	MYL3_RAT	22,142	5.0	11	679	100	605	100
89	D23		Eukaryotic translation initiation factor 5A-1 OS=Rattus norvegicus GN=Eif5a PE=1 SV=3	IF5A1_RAT	16,821	5.1	8	556	100	501	100
90	D24		NADH dehydrogenase [ubiquinone] 1 alpha subcomplex assembly factor 3 OS=Rattus norvegicus GN=Ndufaf	NDUF3_RAT	20,683	7.7	6	175	100	139	100
91	E1		Tubulin-specific chaperone A OS=Rattus norvegicus GN=Tbca PE=1 SV=1	TBCA_RAT	12,736	5.4	4	66	100	53	100
93	E2		Transthyretin OS=Rattus norvegicus GN=Ttr PE=1 SV=1	TTHY_RAT	15,710	5.8	8	646	100	580	100
96	E3		Myosin regulatory light chain 2, ventricular/cardiac muscle isoform OS=Rattus norvegicus GN=Myl2 PE	MLRV_RAT	18,868	4.9	6	232	100	194	100
97	E4		Nuclear transport factor 2 OS=Rattus norvegicus GN=Nutf2 PE=1 SV=1	NTF2_RAT	14,469	5.1	5	84	100	48	100
98	E5		ATP synthase-coupling factor 6, mitochondrial OS=Rattus norvegicus GN=Atp5j PE=1 SV=1	ATP5J_RAT	12,487	9.4	7	417	100	355	100
99	E6		Hemoglobin subunit beta-1 OS=Rattus norvegicus GN=Hbb PE=1 SV=3	HBB1_RAT	15,969	7.9	12	599	100	484	100
100	E7		Hemoglobin subunit beta-1 OS=Rattus norvegicus GN=Hbb PE=1 SV=3	HBB1_RAT	15,969	7.9	13	252	100	124	100
101	E8		Hemoglobin subunit beta-1 OS=Rattus norvegicus GN=Hbb PE=1 SV=3	HBB1_RAT	15,969	7.9	12	418	100	292	100
102	E9		Hemoglobin subunit beta-1 OS=Rattus norvegicus GN=Hbb PE=1 SV=3	HBB1_RAT	15,969	7.9	12	696	100	579	100
103	E10		Hemoglobin subunit beta-1 OS=Rattus norvegicus GN=Hbb PE=1 SV=3	HBB1_RAT	15,969	7.9	13	460	100	331	100

Control

1 fmol	C18		beta-D-galactosidase [Escherichia coli IAI39]	gi|218698747	116,583	5.2	22	445	100	343	100
2 fmol	C19		beta-D-galactosidase [Escherichia coli IAI39]	gi|218698747	116,583	5.2	25	487	100	359	100
5 fmol	C20		beta galactosidase small chain [Escherichia coli MS 107-1]	gi|300817121	116,441	5.3	25	510	100	381	100
10 fmol	C21		beta galactosidase small chain [Escherichia coli MS 107-1]	gi|300817121	116,441	5.3	22	459	100	357	100

high confidence
low confidence
no confidence

Supplementary Table 2. Within group fold changes and p-values for proteins.

Assigned ID	Original No.	Appearance	SED Old / SED Young		EX Old / EX Young	
			P value*	Av. Ratio	P value*	Av. Ratio
1	30	48 (72)	0.7400	-1.0	0.5800	-1.2
2	46	66 (72)	0.9300	1.0	0.6000	1.1
3	95	63 (72)	0.0510	-1.4	0.4600	-1.3
5	109	69 (72)	0.0570	1.3	0.4100	1.1
6	108	66 (72)	0.1800	1.4	0.9700	-1.1
7	132	66 (72)	0.4700	1.0	0.0120	1.2
9	195	66 (72)	0.0580	1.6	0.2600	1.1
13	255	72 (72)	0.0006	-1.8	0.4800	1.1
15	274	69 (72)	0.0022	-1.6	0.3600	1.1
19	346	69 (72)	0.8000	-1.1	0.2300	1.2
23	385	48 (72)	0.0071	1.3	0.6100	1.1
26	424	39 (72)	0.5600	1.2	0.3000	-1.5
27	428	72 (72)	0.0046	-1.5	0.0170	-1.6
28	437	72 (72)	0.0083	-1.5	0.0450	-1.5
29	429	72 (72)	0.7600	-1.1	0.4100	1.1
33	521	72 (72)	0.6200	-1.1	0.3300	-1.1
34	417	51 (72)	0.6000	1.2	0.6200	1.5
39	478	69 (72)	0.0200	-1.9	0.0011	-2.3
40	528	69 (72)	0.0012	2.3	0.0002	2.0
43	581	54 (72)	0.2500	-1.2	0.0050	-1.4
50	701	72 (72)	0.0160	-1.4	0.6500	1.0
52	662	72 (72)	0.0550	2.2	0.1800	1.8
54	735	72 (72)	0.4200	-1.1	0.1100	-1.3
55	748	72 (72)	0.4800	-1.1	0.1500	-1.3
58	903	72 (72)	0.2200	-2.4	0.0045	3.9
59	946	72 (72)	0.0800	1.7	0.0280	-2.0
60	913	72 (72)	0.0001	2.2	0.0160	1.5
61	924	72 (72)	0.0017	2.0	0.1200	1.3
62	927	72 (72)	0.0084	-1.7	0.6600	-1.1
66	1112	72 (72)	0.3700	-1.4	0.1600	-1.6
67	1118	72 (72)	0.2200	-1.6	0.0670	-1.8
68	1257	69 (72)	0.9800	-1.0	0.7100	-1.1
69	1255	72 (72)	0.5500	1.1	0.9100	-1.0
71	1279	72 (72)	0.2400	-1.2	0.7700	1.1
72	1340	72 (72)	0.3600	-1.3	0.1400	-1.2
73	1285	72 (72)	0.7400	-1.0	0.0610	1.7
75	1372	60 (72)	0.8800	1.0	0.1800	-1.3

76	1360	72 (72)	0.0004	1.7	0.0400	1.2
78	1387	72 (72)	0.0240	1.4	0.7600	-1.1
80	1431	72 (72)	0.4200	-1.2	0.4000	1.1
81	1459	72 (72)	0.0280	1.3	0.0030	1.3
83	1484	72 (72)	0.0036	-1.9	0.1100	-1.6
84	1534	69 (72)	0.0073	1.2	0.1100	1.3
85	1531	66 (72)	0.5500	1.1	0.0250	1.5
87	1634	72 (72)	0.1600	1.5	0.9400	-1.2
88	1707	72 (72)	0.0068	2.0	0.0180	2.0
89	1891	72 (72)	0.0041	-1.4	0.6800	1.0
90	1939	72 (72)	0.8300	-1.1	0.4800	-2.1
91	2010	72 (72)	0.0910	1.3	0.2100	-1.2
93	2109	72 (72)	0.0080	-1.6	0.2300	-1.3
96	2191	45 (72)	0.6000	1.0	0.0170	2.0
97	2229	51 (72)	0.8200	-1.1	0.7800	-1.0
98	2260	27 (72)	0.2400	1.2	0.5600	2.2
99	2178	72 (72)	0.1500	-1.6	0.0140	-1.6
100	2183	72 (72)	0.1300	-1.7	0.0200	-1.6
101	2141	72 (72)	0.3100	-1.4	0.0280	-1.7
102	2152	66 (72)	0.4500	-1.4	0.0073	-1.9
103	2150	60 (72)	0.3000	-1.4	0.0400	-1.5

*P value indicates < 0.05
